# Phlebitis and infiltration: vascular trauma associated with the peripheral venous catheter

**DOI:** 10.1590/1518-8345.2377.3002

**Published:** 2018-05-17

**Authors:** Luciene Muniz Braga, Pedro Miguel Parreira, Anabela de Sousa Salgueiro Oliveira, Lisete dos Santos Mendes Mónico, Cristina Arreguy-Sena, Maria Adriana Henriques

**Affiliations:** 1 MSc. Doctor degree student. Universidade de Lisboa, Instituição, Lisboa, Portugal, Portugal. Adjunct Professor. Departamento de Medicina e Enfermagem, Universidade Federal de Viçosa, Viçosa, MG, Brazil. Coordenação de Aperfeiçoamento de Pessoal de Nível Superior-CAPES . Proceso Número 0867/14-4.; 2 PhD. Adjunct Professor. Unidade Científico-Pedagógica de Enfermagem Fundamental, Escola Superior de Enfermagem de Coimbra, Coimbra, Portugal, Portugal.; 3 PhD. Adjunct Professor. Unidade Científico-Pedagógica de Enfermagem Fundamental, Escola Superior de Enfermagem de Coimbra, Coimbra, Portugal, Portugal.; 4 PhD. Assistant Professor.Unidade, Universidade de Coimbra, Coimbra, Portugal, Portugal.; 5 PhD. Associated Professor. Departamento de Enfermagem Aplicada, Universidade Federal de Juiz de Fora, Juiz de Fora, MG, Brazil.; 6 PhD. Adjunct Professor. Unidade, Escola Superior de Enfermagem de Lisboa, Lisboa, Portugal, Portugal.

**Keywords:** Nursing, Catheterization Peripheral, Phlebitis, Infusions Intravenous, Extravasation of Diagnostic and Therapeutic Materials, Patient Safety

## Abstract

**Objective:**

to determine the incidence rate and risk factors for the nursing-sensitive indicators phlebitis and infiltration in patients with peripheral venous catheters (PVCs).

**Method:**

cohort study with 110 patients. Scales were used to assess and document phlebitis and infiltration. Socio-demographic variables, clinical variables related to the PVC, medication and hospitalization variables were collected. Descriptive and inferential analysis and multivariate logistic models were used.

**Results:**

the incidence rate of phlebitis and infiltration was respectively 43.2 and 59.7 per 1000 catheter-days. Most PVCs with these vascular traumas were removed in the first 24 hours. Risk factors for phlebitis were: length of hospital stay (p=0.042) and number of catheters inserted (p<0.001); risk factors for infiltration were: piperacillin/tazobactan (p=0.024) and the number of catheters inserted (p<0.001).

**Conclusion:**

the investigation documented the incidence of nursing-sensitive indicators (phlebitis and infiltration) and revealed new risk factors related to infiltration. It also allowed a reflection on the nursing care necessary to prevent these vascular traumas and on the indications and contraindications of the PVC, supporting the implementation of the PICC as an alternative to PVC.

## Introduction

Nursing care at the hospital is essential for health prevention and promotion, patient safety and restoration of health and well-being. This includes care related to the insertion of peripheral venous catheters (PVCs), and their maintenance and monitoring^1^ ^-^ ^2^. 

Evidence shows that 58.7% to 86.7% of patients have a venous catheter inserted during their hospitalization, so PVCs are a significant portion of the care delivered by nurses^3^ ^-^ ^5^ ^).^ PVCs have become an indispensable resource for hospital care, necessary for intravenous administration of medications, solutions, blood components, parenteral nutrition and also for diagnostic purposes^4^ ^,^ ^6^ ^-^ ^7^. However, these devices are not free of complications. Several studies have documented a high incidence of peripheral vascular trauma associated with the use of PVCs, including phlebitis and infiltration^7^ ^-^ ^11^.

Phlebitis is an inflammation in the intimal layer of the vein, developed in response to tissue damage caused by factors associated with the insertion and use of the PVC and the medications administered through it. It is identified by clinical manifestations such as pain, erythema, blushing, edema and palpable venous cord^12^ ^-^ ^13^. Studies assessing the incidence of phlebitis found values between 1.2% and 54.5%^7^ ^,^ ^14^ ^-^ ^17^. The studies indicate factors related to the characteristics of the patient, of the PVC and of the medications administered as risk factors for the development of phlebitis^14^ ^-^ ^15^ ^,^ ^17^. 

Infiltration is another type of vascular trauma, resulting from a lesion in the layers of the vein and a subsequent perforation, leading to infiltration of non-vesicant solutions or medications in the tissues surrounding the catheter insertion site. When the solutions or medications have vesicant properties, the leakage is called extravasation^18^ ^-^ ^19^. Edema is the most frequent clinical sign of infiltration, and it may be associated with others such as pale skin, pain, temperature decrease and/or sensitivity at the site. More severe cases of infiltration may also lead to circulatory impairment and tissue necrosis^8^ ^,^ ^18^ ^,^ ^20^. The incidence of infiltration ranges from 7% to 40.5%^2^ ^,^ ^8^ ^,^ ^16^. The risk factors described in the literature are based on case reports or series of cases, and are mainly related to the medications administered through the PVC, such as: dopamine, beta blockers/adrenaline, calcium gluconate, isotonic glucose solution, potassium, parenteral nutrition, sodium bicarbonate, various types of antimicrobials and chemotherapeutic drugs and solutions^18^ ^,^ ^20^ ^-^ ^22^.

This article warns us about the risk of peripheral vascular trauma associated with the use of PVC and points out the need to increase the evidence on nursing-sensitive quality indicators, namely for the incidence of phlebitis and infiltration and the possible risk factors for these complications, with the objective of producing knowledge and implementing evidence-based practices in nursing care. Aiming at improving the quality of nursing care and patient well-being, this study was carried out to determine the incidence rate and risk factors for nursing-sensitive indicators - phlebitis and infiltration - in patients using PVCs.

## Method

A descriptive cohort study was carried out in the medical clinic of a hospital in the central region of Portugal. The choice for this unit was based on the results of nursing-sensitive indicators, namely the incidence of phlebitis (43.8%) and infiltration (13%), evidenced in this unit in 2012^14^ and on the need to evaluate these results over time. Another reason for this choice was the motivation of the nursing team to get to know the results of their practices, which in turn could support reflection and implementation of evidence-based practices to improve patient care.

The non-probability sample included 121 patients admitted to the unit between July 10^th^ and September 10^th^, 2015, who met the following inclusion criteria: age ≥ 18 years and having one or more PVCs. Twelve patients were excluded (four patients with CVC, three who refused and four who did not sign the Consent Form). Thus, the sample consisted of 110 patients who used one or more PVCs, totaling 526 PVCs (1389 catheter-days).

 It should be mentioned that new patients were allowed to enter the cohort and there were no follow-up losses. On the last day of the study (September 10^th^) there were 28 patients on intravenous therapy. In order to evaluate the entire period of treatment, the 28 patients were followed until the end of their intravenous treatment, totaling 82 days of follow-up. When the patient had more than one PVC inserted, all of them were considered for statistical analysis. 

Socio-demographic variables (age and gender), hospitalization characteristics (reason and time of hospitalization), clinical variables (initial diseases) and variables related to medications administered in the PVC were obtained from the electronic patient record. The variables related to the PVC that were not available in the patient’s chart were collected through the evaluation of the insertion and removal site by the nurses in the unit and by the main researcher. These variables are: duration (hours), gauge (G), number of venipuncture attempts, insertion site, type of dressing, and signs and symptoms of phlebitis and infiltration. The insertion of each PVC was considered a new case and the patients were followed from hospitalization to discharge, transference or death.

The 27 nurses who provided direct care to the patients were previously and individually trained by the investigator to evaluate the PVC insertion site regarding the presence of signs and symptoms of phlebitis and infiltration. The Portuguese Scales of Phlebitis and Infiltration were used to standardize the evaluation and registration of the signs and symptoms^13^ ^,^ ^19^. In addition, in order to avoid information bias, absence of data and potential influence of nurses in the results, the researcher evaluated the insertion site and the removal of PVCs looking for signs and symptoms of phlebitis and infiltration before the end of each nurse work shift (morning, afternoon and night). In addition, the researcher directly consulted the nurses about the replacement of the PVCs and, if necessary, compared the nurses’ records with the clinical manifestations presented by the patient. It should be mentioned that there was no divergence between the evaluation and the records made by the nurses and by the researcher regarding the presence of phlebitis and infiltration.

In order to reduce the risk of bias on the grade of phlebitis and infiltration, only the signs and symptoms were available in the data collection instrument. Subsequently, the investigator converted the signs and symptoms to the respective grades of phlebitis and infiltration. 

The data obtained were analyzed with the software Statistical Package for the Social Sciences (SPSS) version 20,0 (IBM SPSS, Chicago). Descriptive statistics (absolute and relative frequencies), measures of central tendency (mean and median) and dispersion (interquartile values, standard deviation, minimum and maximum values) were used, followed by inferential statistics.

In order to evaluate the possible risk factors associated with the dichotomous variables phlebitis and infiltration (0 =no; 1 =yes) a point-biserial correlation analysis was conducted between phlebitis and infiltration and the continuous variables patient age, length of hospital stay, number of catheters inserted, number of venipunctures, duration of catheter, number of administrations of antimicrobials, and number of administrations of other medications. The phi correlation coefficient was used to assess the existence of associations with the nominal variables expressed as frequency (gender, reason for hospitalization, initial diseases, insertion site, dressing used to secure the catheter and medication administered), and the correlation coefficient ρdr was used between phlebitis and infiltration and the ordinal variable (*rank*) catheter gauge^23^. These analyzes allowed to select the predictor variables with statistically significant correlation with the presence of phlebitis and infiltration. 

Then, a hierarchical multivariate logistic regression analysis was conducted with the predictor variables resulting from the association tests and the dependent variables phlebitis and infiltration. The model was adjusted to maintain only the predictor variables with type I error in the final logistic model (p < 0.05). The Hosmer-Lemeshow test was used to verify the quality of fit. The Area Under Curve (AUC) analysis of the Receiver Operating Characteristic Curve (ROC Curve) was used to assess the discriminant capacity of the model used. 

The analysis of incidence rate considered the quotient between the number of catheters with the outcome (phlebitis or infiltration) and the total number of days of venous catheter use in the period per thousand. The cumulative incidence considered the quotient between the number of catheters that presented the outcome (phlebitis or infiltration) and the total number of catheters in the period, multiplied by 100^24^ ^).^

The research followed all ethical considerations for research involving human subjects and was approved by the Ethics Committee of the Hospital (Ref. 020-15).

## Results

Half of the patients were women (52.7%), with a mean age of 79 years (18-96, SD±13.0) and a median age of 82 years (Q1=77.0, Q3=86.0). Hypertension (60.9%) and metabolic pathologies (48.2%) were the most common pre-existing diseases. Infectious disease was the main cause of hospitalization (72.7%).

Five PVCs were inserted on average in each patient during the entire treatment (1-20; *SD*±3.6), with a mean of 1.5 venipuncture attempts before successful insertion of PVC (1-8; *SD*±0.8) and a median of one puncture in 80% of the cases (Q1=1.0; Q3=1.0). During the entire hospitalization, the mean number of punctures in each patient was 6.5 (1-49; *SD*±6.5), with a median of four punctures (Q1=2.0; Q3=8.0). The insertion site of the PVCs was mainly the back of the hand (39.7%) and the forearm (35.4%), with the gauges 22G (59.9%) and 20G (37.3%). The most widely used dressing was sterile transparent film (88.8%). Table 1 presents the characterization of patients regarding age in age group, use of PVC and the main drugs administered through the PVC.

**Table 1:** Characterization of patients regarding age, use of peripheral venous catheter and medications administered. Coimbra, PT, 2015

Variables	*n*	%
Age group in years (N=110 patients)		
18 - 34	2	1.8
35 - 49	3	2.7
50 - 64	3	30.0
65 - 79	68	61.8
≥ 80		
Catheter insertion site (N=526 PVC^†^)		
Back of the hand	209	39.7
Antecubital fossa	55	10.4
Forearm	186	35.4
Arm	49	9.4
Lower limb - Foot	27	5.1
Catheter gauge (N=526 PVC^†^)		
≤ 18G	12	2.3
20G	196	37.3
22G	316	60.0
24G	2	0.4
Number of venipuncture attempts (N=526 PVC^†^)		
1 puncture	422	80.2
2 punctures	53	10.1
3 punctures	33	6.3
4 a 8 punctures	18	3.4
Dressing used to secure catheter (N=526 PVC^†^)		
Non-sterile white plaster	59	11.2
Sterile transparent film	467	88.8
Medications administered (N=110 patients)*		
Antacid	55	50.0
Antiarrhythmic	6	5.5
Antimicrobial	95	86.3
Bronchodilator	2	1.8
Corticosteroid	3	2.7
Diuretic	64	58.2
Continuous intravenous solution	89	80.9

Note: *The percentage does not correspond to 100% because this variable presents multiple answers; ^†^PVC - Peripheral Venous Catheter.

The incidence rate of phlebitis and infiltration was respectively 43.2 and 59.7 per thousand catheter-days and the cumulative incidence per catheter was respectively 11.5% and 15.8%. Grade 4 phlebitis and grades 3 and 4 infiltration were not found. The presence of post-infusion phlebitis was not assessed. The mean duration of PVCs in the patients was 61.1h, that is, 2.5 days (1-528h;*SD*±66.7), with a median of 38h (Q1=23.0; Q3=73.0). PVCs that did not result in complications, that is, those that were removed due to end of treatment or discharge (*M=*86.5h;*SD*±79.1) took significantly longer to be removed than the PVCs removed due to complications (*M=*55h;*SD*±62.0;*t*(136.261)=-3.770; *p*<0.001). The mean duration of the 60 PVCs removed due to phlebitis was 83.5h (8-528; *SD*±101.3), with a median of 38h (Q1=24.0; Q3=107.0). For PVCs with infiltration, the mean duration was 40.5h (1-195;*SD*±35.4), with a median of 28h (Q1=19.0; Q3=48.0). Table 2 presents the characterization of the duration of the PVCs according to the reason for removal (phlebitis or infiltration) and the respective grades.

**Table 2:** Characterization of the duration of the peripheral venous catheter until removal due to phlebitis or infiltration and the respective grade. Coimbra, PT, 2015

	Phlebitis (****n**** =60)		Infiltration (****n**** =83)	
Variables	*n*	%	*n*	%
Duration of catheter*				
Less than 24h	18	30.0	37	44.6
25 to 48h	17	28.3	29	35.0
49 to 72h	7	11.8	5	6.0
73 to 96h	3	5.0	5	6.0
97 to 120h	2	3.3	5	6.0
121 to 168h	5	8.3	1	1.2
More than 169h	8	13.3	1	1.2
Grade				
Grade 1	38	63.5	70	84.5
Grade 2	15	25.0	13	15.5
Grade 3	7	11.5	-	-
Grade 4	-	-	-	-

Note: *The mean duration of the PVC was 61.1h (1-528h; *SD*±66.7).

According to the multivariate logistic model, the variables that presented a statistically significant influence on the Logit of the probability of the patient presenting phlebitis were the length of hospital stay (p=0.042) and the number of catheters inserted (p<0.001). Specifically, an increase of one day in the length of hospital stay increased the probability of phlebitis by 1.07 times, and an increase of one PVC in the patient increased the probability of phlebitis by 1.37 times. The Hosmer-Lemeshow test (p=0.549) revealed a good fit in the model, which correctly classified 77.5% of the cases (p<0.001), showed a sensitivity of 54% and a specificity of 90%, as well as a good discriminant capacity (AUC=0.816; p<0.001; CI 95% [0.735-0.897]). 

For the outcome infiltration, the variables most likely to be risk factors were the antibiotic piperacillin/tazobactam (p=0.024) and the number of catheters inserted (p<0.001). The probability of infiltration in the patient who received piperacillin/tazobactam through the PVC was 3.65 times higher than in patients who did not use this antibiotic. For each addition in the number of PVCs in the patient, the probability of infiltration increased 1.45 times. According to the Hosmer & Lemeshow test, the model was not a good fit to the data (p=0.044); however, it correctly classified 78% of the cases (p<0.001), showed a sensitivity of 68% and specificity of 86.7%, as well as good discriminant capacity (AUC=0.837; p<0.001; CI 95% [0.762-0.912]). Table 3 presents the variables that had a higher probability of being a risk factor for phlebitis and infiltration and the respective values ​​of odds ratio (OR) and p-value.

**Table 3:** Logit coefficients of the multivariate logistic regression model of the outcomes phlebitis and infiltration. Coimbra, PT, 2015

Variables	ß*	SE^**†**^	OR^**‡**^	CI^**§**^ [95%]	*X* ^***2***^ ****Wald**** ^***ǁ***^	p*-*value
Phlebitis						
Length of hospital stay	0.06	0.03	1.07	[1.00-1.14]	4.153	0.042
Number of catheters inserted	0.31	0.08	1.37	[1.15-1.63]	12.258	<0.001
Infiltration						
Piperacilin/tazobactam	1.29	0.57	3.65	[1.18-11.25]	5.079	0.024
Number of catheters inserted	0.37	0.09	1.45	[1.21-1.71]	16.761	<0.001

Note: *ß= beta; †SE = standard error; ‡OR = *odds ratio*; §CI [95%] = 95% confidence interval; ^||^ *X* ^2^ *Wald.*

## Discussion

The assessment of the nursing-sensitive quality indicator incidence of phlebitis in 110 patients with a PVC showed a cumulative incidence of 11.5%. This result is in agreement with other studies, which found values between 10.1% and 43.0%^9^ ^-^ ^11^ ^,^ ^16^ ^,^ ^25^. The current rate (11.5%) represents a significant reduction when compared to the incidence of phlebitis found previously in this unit (43.8%)^14^. However, it still exceeds the 5% recommended by the Infusion Nurses Society^11^. This difference in incidence may be associated with the implementation of new evidence-based practices in nursing care after the action-research carried out in the unit between 2012 and 2014 (substitution of non-sterile dressings for semipermeable and sterile dressings in the insertion site, indication for selecting the smallest PVC gauges and use of disinfected tourniquets, among other practices)^14^. Another difference may be due to the different scales used to evaluate the signs and symptoms of phlebitis and its grades.

The infiltration was another outcome analyzed. It presented clinical and epidemiological importance due to the cumulative incidence of 15.8% found in this investigation. This result is lower than other studies with rates of 23% and 31.5%^9^ ^-^ ^10^ and higher than the incidences of infiltration of 7% and 13% found in studies conducted in Portugal^14^ ^,^ ^16^. This difference may be due to the use of a scale^19^ to evaluate the signs and symptoms of infiltration in the present study, reducing variability in documentation and information bias. A standardized evaluation of this indicator was not assured in other studies^14^ ^,^ ^16^.

It should be mentioned that the differences between the studies regarding the incidence of phlebitis and infiltration may also be due to the characteristics of the patients in the sample and the limitations particular to each study.

The risk factors for phlebitis revealed in this study were the length of hospital stay and the number of catheters inserted in the patients, which are the same as those reported in a study carried out in Spain^25^. However, these risk factors were not evidenced in other studies^14^ ^,^ ^16^ ^-^ ^17^ ^,^ ^26^. 

The risk factors for the occurrence of infiltration were the antibiotic piperacillin/tazobactam and the number of catheters inserted in the patient. These risk factors were not identified in other studies, which have a low evidence level, since they are based on case reports and series of cases and have small samples^18^ ^,^ ^20^ ^-^ ^22^. Only a retrospective study with children used logistic regression to assess the risk factors for infiltration, evidencing as risk factors insertion in the lower limbs, hospitalization in pediatrics and administration of medication^27^. 

The clinical manifestations of phlebitis and infiltration were identified by the nurse mainly in the first 72 hours after insertion of the PVC (70.1% and 85.6%, respectively) and with a higher percentage in the first 24 hours. This result is in agreement with the period for manifestation of phlebitis and infiltration found in other studies^9^ ^,^ ^11^ ^,^ ^16^ ^-^ ^17^. In addition, this reinforces the importance of removing the PVC when the first signs and symptoms are identified, and not according to a defined period of time. These results also emphasize the importance of frequent inspection of the PVC insertion site and surrounding areas by the nurse, who should use validated scales in order to standardize the evaluation of the insertion site and surrounding areas, support decision-making and improve the documentation and analysis of the grade of the problem^13^ ^,^ ^19^. It also indicates the need to include the participation of the patient and/or family members in the care^28^, aiming to identify early signs and symptoms of peripheral vascular trauma and improve the quality of care. Pain in the PVC insertion site and surrounding areas is one of the first signs of phlebitis and infiltration, present in their 1^st^ grade^13^ ^,^ ^19^. Early identification of pain and removal of the PVC for this reason may interrupt the progression of the inflammatory process to clinical manifestations with deeper tissue involvement. 

In order to improve the quality of nursing care and prevent the occurrence of phlebitis and infiltration, the nurse should analyze the characteristics of the patient, the intravenous medications prescribed (irritant and/or vesicant, pH and osmolarity), the expected duration of the intravenous treatment and the risk factors for the occurrence of these complications before selecting a venous catheter. In addition, the nurse should evaluate the risks and benefits of each type of catheter and consider the patient’s preferences^6^. This analysis may indicate other venous catheters to the patient, such as peripherally inserted central catheters(PICC)^6^.

The limitations of the present investigation are the data related to a single unit, the size of the sample and the non-probabilistic sampling, limiting the generalization of the results. Another limitation was the lack of evaluation of phlebitis after the removal of the PVC. 

Despite the limitations, the results of the present study broaden the knowledge about the risk factors for the occurrence of infiltration in adult patients using PVC for intravenous drug administration. In addition, the feedback of the results to the Nursing team provided a reflection on the nursing-sensitive quality indicators related to phlebitis and infiltration and their respective risk factors. It also allowed a reflection on the nursing care needed to prevent these vascular traumas and indications and contraindications of the PVC. This supported the implementation of the PICC as an alternative to PVC. The results of the use of the PICC in the patients of this institution have been object of investigation.

## Conclusion

This study allowed the documentation of the results of nursing-sensitive quality indicators (phlebitis and infiltration) related to peripheral venous catheterization for administration of intravenous drugs. In addition, it revealed new risk factors related to the occurrence of infiltration in adult patients with PVC. 
